# Relationships Between Psychological Resilience, Rejection Sensitivity, and Short-Video Addiction in University Students: A Cross-Lagged Analysis

**DOI:** 10.3390/bs16071238

**Published:** 2026-07-21

**Authors:** Yang Liu, Min Xie, Shuyue Zhang

**Affiliations:** 1 Faculty of Education, Guangxi Normal University, Guilin 541004, China; 2 School of Marxism, Beibu Gulf University, Qinzhou 535011, China; 3 Guangxi College and University Key Laboratory of Cognitive Neuroscience and Applied Psychology, Guangxi Normal University, Guilin 541004, China

**Keywords:** short-video addiction, psychological resilience, rejection sensitivity, cross-lagged analysis, I-PACE model, behavioral addiction

## Abstract

Short-video addiction has emerged as a growing behavioral concern in the context of the widespread use of platforms such as Douyin and TikTok, posing potential risks to young adults’ psychological well-being. Psychological resilience and rejection sensitivity are considered important psychological characteristics that may function as protective and risk factors, respectively, in the development of addictive media use. However, the longitudinal interplay among these variables remains insufficiently understood. This study examined the longitudinal relationships among psychological resilience, rejection sensitivity, and short-video addiction over time among university students. A two-wave longitudinal design with a six-month interval was conducted among 1394 Chinese university students. Participants completed validated measures assessing short-video addiction, psychological resilience (CD-RISC-10), and rejection sensitivity (RSQ). Cross-lagged panel modeling (CLPM) was employed to investigate the bidirectional predictive associations among these variables while controlling for autoregressive effects. All three constructs demonstrated substantial temporal stability across the two measurement waves. Cross-lagged analyses indicated that higher psychological resilience at Time 1 predicted lower rejection sensitivity and lower short-video addiction at Time 2, whereas higher rejection sensitivity at Time 1 predicted lower psychological resilience and higher short-video addiction over time. Although these longitudinal associations were statistically significant, their effect sizes were modest. These findings suggest that psychological resilience and rejection sensitivity may represent protective and vulnerability-related psychological characteristics associated with short-video addiction over time. The findings support the I-PACE model by highlighting the dynamic and longitudinal associations among psychological resources, interpersonal vulnerability, and addictive digital behaviors. Interventions aimed at strengthening psychological resilience and reducing rejection sensitivity may contribute to preventing short-video addiction and promoting healthier digital engagement among university students.

## 1. Introduction

With the rapid development and deep penetration of mobile internet technologies, short-video platforms represented by Douyin and TikTok are profoundly reshaping the ways people acquire information and engage in social interaction. Characterized by fragmentation, rapid updates, and strong sensory stimulation, this form of content not only enhances entertainment and information delivery efficiency but also introduces a new risk of behavioral addiction short-video addiction. Compared with traditional internet addiction or social media addiction, short-video addiction displays distinct features: its use pattern is higher in frequency and lower in cost, with a more powerful reinforcement loop that swiftly activates the brain’s reward system and fosters persistent usage inertia, thereby exerting deeper erosion on individuals’ cognitive resources, emotional regulation, and goal maintenance functions (Y. Wang & Yuan, 2025).

Short-form video platforms produce a distinct pattern of use—high frequency, rapid reward, and algorithmic personalization—that has been empirically linked to addictive-like symptoms and adverse cognitive and affective outcomes. Recent longitudinal and neuroimaging studies indicate that short-video overuse is associated with depressive symptoms, impaired attention, altered reward processing, and riskier decision making, underscoring its emerging public-health significance (Y. Qin et al., 2022).

Previous studies have shown that short-video addiction is positively associated with psychological problems such as anxiety and depression; its tolerance demands and anhedonia are covariant, which further aggravates mental-health risks (Qu et al., 2024). Against this backdrop, psychological resilience, defined as an individual’s capacity to cope with stress, withstand adversity, and maintain psychological homeostasis, has attracted increasing attention. Existing research has revealed a negative association between resilience and both internet or social-media addiction. Resilience not only buffers the detrimental impact of addictive behaviors on mental health but also moderates the relationship between loneliness and such behaviors (Zhou et al., 2025). Longitudinal network and cross-lagged studies have begun to unpack temporal relationships between short-video addiction and mental-health indicators (e.g., depression), revealing specific symptom pathways such as tolerance and anhedonia that carry forward over time (Yam et al., 2024). These methods help move beyond cross-sectional correlation to identify potential directional effects.

Nevertheless, research specifically targeting short-video addiction remains limited, especially concerning whether a unique mechanism exists between psychological resilience and short-video addiction, which warrants further investigation. Meanwhile, rejection sensitivity, as an important interpersonal psychological trait, may play a key role in this process. On the one hand, resilience can alleviate rejection sensitivity by strengthening positive cognition and emotional regulation, thereby reducing perceived threats in social interactions (C. Xu et al., 2025). On the other hand, individuals with high rejection sensitivity are more likely to seek emotional compensation through virtual interactions when facing setbacks in real-life social contexts; the immediate feedback and gratification provided by short-video platforms may intensify this dependence and lead to addictive risk (Zhang et al., 2024).

However, few studies have systematically examined the role of rejection sensitivity in the relationship between psychological resilience and short-video addiction, and the potential mediating or moderating effects remain insufficiently tested. Therefore, this study employs a cross-lagged panel design to explore the dynamic relationships among psychological resilience, rejection sensitivity, and short-video addiction in a university student population. The findings are expected to clarify the unique psychological processes distinguishing short-video addiction from other addictive behaviors, deepen understanding of the mechanisms by which resilience and rejection sensitivity operate in this context, and provide theoretical and practical implications for developing targeted interventions.

### 1.1. Psychological Resilience and Short-Video Addiction

A growing body of research has demonstrated that psychological resilience is an important protective factor against internet and social-media addiction. Psychological resilience refers to an individual’s ability to adapt effectively and recover from adversity or stressful circumstances (Newman, 2005). Within studies of internet addiction, resilience has been shown not only to be significantly and negatively associated with the severity of addiction (Kutuk, 2023; Li et al., 2019) but also to fully mediate the relationship between internet addiction and anxiety (Yue et al., 2020). In addition, higher levels of resilience help individuals with low perceived social support overcome tendencies toward smartphone addiction (Qiu et al., 2020), significantly predict the severity of social-media addiction (Bilgin & Taş, 2018; Öztürk & Kundakçı, 2021; Shen, 2020; Wu et al., 2024), and buffer the negative impact of smartphone addiction on sleep quality (Xie et al., 2023). For adolescents with high levels of loneliness, resilience can also reduce the risk of social-media addiction (Yam et al., 2024). Recent cross-cultural and longitudinal work indicates that psychological resilience consistently functions as a protective factor against internet and smartphone addictive behaviors across diverse samples; some studies show resilience predicts lower addictive behaviors prospectively and mediates relationships between stressors and problematic use (Almulla et al., 2025). Collectively, these findings suggest that resilience plays a crucial protective role in the formation and development of addictive behaviors.

However, most existing studies have focused on internet or social-media addiction, and evidence regarding the association between psychological resilience and short-video addiction remains relatively scarce. Preliminary studies have reported that generalized internet and social-media addictions are significantly related to lower resilience and poorer mental-health outcomes (Hao et al., 2023; Mak et al., 2018); moreover, resilience has been found to moderate the relationship between loneliness and short-video addiction (Zhou et al., 2025). Nevertheless, the majority of such studies have adopted cross-sectional designs, making it difficult to clarify the causal direction between resilience and short-video addiction.

Although short-video addiction shares some similarities with internet and social-media addiction in terms of addictive features and psychological consequences, it also presents notable differences. Short-video platforms are characterized by high frequency of use, immediate feedback, and algorithm-driven recommendations, which more readily induce user immersion and behavioral inertia, leading to more pronounced learning interruptions, anxiety, and other adverse psychological outcomes (Mao & Liao, 2025; Y. Qin et al., 2022). Whether the relationship between psychological resilience and short-video addiction exhibits a dynamic pattern distinct from that of other addictive behaviors remains to be verified, and longitudinal research is needed to more clearly elucidate their association.

### 1.2. Rejection Sensitivity and Short-Video Addiction

Rejection sensitivity refers to an individual’s heightened vigilance and emotional reactivity to potential exclusion or rejection in social interactions (Downey & Feldman, 1996). It is typically characterized by a tendency to interpret ambiguous or neutral social cues as rejection, accompanied by intense negative emotions and avoidant behaviors (Downey & Feldman, 1996). Previous studies have shown that rejection sensitivity is positively associated with internet and social-media addiction among adolescents; that is, individuals with higher rejection sensitivity are more likely to become excessively engaged in online or social-media use (Kuss & Griffiths, 2017). This may be because individuals high in rejection sensitivity rely more heavily on virtual environments to obtain emotional compensation and social approval, thereby increasing the risk of problematic use.

In addition, people with high rejection sensitivity often display pronounced avoidance and defensive tendencies in real-life social interactions (Downey & Feldman, 1996; Romero-Canyas et al., 2010), making it difficult for them to gain stable support and a sense of belonging through offline relationships. Such deficits in social functioning may prompt them to use short videos as a substitute for social connection and emotional regulation, leading to a “negative cycle”: rejection sensitivity fosters excessive reliance on short-video platforms, while addiction to short videos may further erode real-world social skills and mental health, thereby intensifying rejection sensitivity (Zhang et al., 2024). Prior research has also indicated that rejection sensitivity not only directly predicts the severity of internet addiction but may also increase addictive risk indirectly by heightening loneliness and reducing perceived social support (Öztürk & Kundakçı, 2021). Several recent studies emphasize rejection sensitivity as a risk factor that predicts problematic internet and social media use, often via increased loneliness or diminished self-control; longitudinal evidence suggests RS increases subsequent problematic use in student samples (Tao et al., 2022).

Overall, existing evidence provides useful insights regarding the link between rejection sensitivity and short-video addiction, but most studies are based on cross-sectional data and cannot capture their longitudinal influences over time. Given the instant feedback and algorithmic recommendations that characterize short-video platforms, rejection sensitivity may exert a more persistent and complex influence on the emergence and maintenance of addictive behaviors. Clarifying the temporal dynamics and potential pathways of this influence requires longitudinal designs to elucidate the unique psychological mechanisms through which rejection sensitivity contributes to the development of short-video addiction.

### 1.3. Rejection Sensitivity, Psychological Resilience, and Short-Video Addiction

In studies on addictive behaviors, psychological resilience and rejection sensitivity are often regarded as two closely related but potentially oppositely directed psychological traits. Psychological resilience reflects an individual’s ability to maintain emotional stability and adaptive coping in the face of adversity, whereas rejection sensitivity represents a heightened alertness and emotional reactivity to potential negative feedback in social interactions. Psychological resilience refers to an individual’s capacity to maintain positive adaptation and recover from adversity and stressful situations (Bonanno & Diminich, 2013; Connor & Davidson, 2003). Previous research has shown that individuals with higher levels of resilience tend to exhibit lower rejection sensitivity; they are more capable of regulating negative emotions through strategies such as cognitive reappraisal and positive attention, thereby reducing excessive processing of rejection cues (Downey & Feldman, 1996; Hobfoll, 1989; Romero-Canyas et al., 2010).

In contrast, individuals with high rejection sensitivity are often in a state of chronic vigilance toward social threats. According to the process model of emotion regulation (Gross, 2015), individuals with high psychological resilience are more likely to regulate emotions at the early stages of emotional generation, such as by reinterpreting the meaning of social situations to reduce unnecessary emotional arousal. This proactive and anticipatory regulation strategy can weaken the sustained vigilance and defensive responses induced by rejection sensitivity, thus reducing anxiety, hostility, and the risk of social media addiction. However, emotional regulation resources may become depleted over time, thereby weakening resilience (Geng et al., 2022; Navarro et al., 2018). The conservation of resources theory posits that under prolonged stress, individuals’ core psychological resources gradually become exhausted (Hobfoll, 1989). When emotional regulation resources are insufficient, individuals’ ability to cope with interpersonal conflicts, frustrations, or stressful events decreases significantly, which in turn undermines their level of resilience. Furthermore, high rejection sensitivity is often accompanied by avoidant attachment styles and negative attributional patterns, which not only limit the effective use of social support systems but also impair individuals’ capacity for emotional recovery and cognitive restructuring (Romero-Canyas et al., 2010).

Based on the I-PACE model (Brand et al., 2016, 2019), the development and maintenance of addictive behaviors typically result from the dynamic interaction among individual predispositions (e.g., rejection sensitivity), emotional and cognitive regulation capacities (e.g., psychological resilience), and situational factors (e.g., the instant feedback mechanisms of short-video platforms). Within this framework, rejection sensitivity and psychological resilience can be conceptualized as risk and protective factors, respectively, while the immediate feedback and algorithmic recommendation systems of short-video platforms constitute significant external stimuli. Psychological resilience has been proposed as a potential mechanism linking rejection sensitivity and problematic digital media use in previous research, although the temporal relationships among these constructs remain insufficiently understood. Therefore, longitudinal research is needed to clarify their directional associations over time. Therefore, longitudinal research could further elucidate the dynamic trends of rejection sensitivity in relation to psychological resilience and short-video addiction.

### 1.4. The Present Study

The present study aimed to investigate the longitudinal relationships among short-video addiction, psychological resilience, and rejection sensitivity. To achieve this goal, we employed a two-wave longitudinal design with a 6-month interval and conducted cross-lagged analyses to examine the bidirectional predictive effects among the variables. This approach allows for a simultaneous examination of how short-video addiction predicts subsequent psychological resilience and rejection sensitivity, as well as how these two psychological factors, in turn, predict changes in short-video addiction. In doing so, the longitudinal associations among these constructs can be better understood.

In the first wave of data collection, participants’ levels of short-video addiction, psychological resilience, and rejection sensitivity were assessed. Six months later, in the second wave, the same instruments and procedures were used to reassess these variables. Through cross-lagged modeling, we were able to control for the stability of each variable while testing the potential causal pathways and directional effects. This research design contributes to a deeper understanding of the complex temporal associations between short-video addiction and psychological adaptation characteristics and provides empirical evidence for developing targeted psychological interventions and media-use management strategies.

## 2. Methods

### 2.1. Participants

The first wave of the survey was conducted in April 2024 among undergraduate students from eight universities in Guangxi Province, China. After excluding questionnaires with irregular response patterns or abnormally short completion times, a total of 1785 valid responses were obtained, yielding an effective response rate of 92.29%. Among these participants, 749 were male and 1036 were female, with a mean age of 20.26 years (SD = 0.81).

The second wave of data collection (T2) took place six months later, in November 2024. During this follow-up, 391 participants from the first wave did not complete the survey (attrition rate = 21.91%), resulting in a final valid sample of 1394 participants (806 females and 588 males), with a mean age of 20.14 years (SD = 0.70).

All questionnaires were administered and collected online via the “Wenjuanxing” platform. Data collection was carried out in classroom settings through a group-based administration approach, with the assistance of class instructors who organized students by class order. Before completing the survey, researchers provided participants with detailed information about the study’s purpose and procedures, emphasizing that all responses would remain strictly confidential and be used solely for academic research. Participants were informed that they could withdraw from the study at any time. Completion of the questionnaire took approximately 5 min. This study was reviewed and approved by the Academic Ethics Committee of the School of Education Science, Guangxi Normal University (Approval No. 2023-012).

### 2.2. Measures

#### 2.2.1. Short Video Addiction Scale

In the present study, the Short Video Addiction Scale was employed to assess participants’ level of addictive use of short video platforms (H. Qin et al., 2019). This instrument was adapted from Leung’s Mobile Phone Addiction Index (MPAI) and consists of 14 items categorized into four subscales: (1) Withdrawal symptoms (5 items; e.g., “Feeling uneasy or irritable without access to short videos”); (2) Emotional escapism (3 items; e.g., “Watching short videos to cope with feelings of loneliness”); (3) Impaired self-control (4 items; e.g., “Repeatedly failing to reduce the time spent on short videos”); and (4) Reduced efficiency (2 items; e.g., “Short video use lowers work or study productivity”). Each item is rated on a five-point Likert scale (1 = strongly disagree, 5 = strongly agree), yielding a total score ranging from 14 to 70. Higher scores indicate a greater degree of short video addiction. In this study, the overall internal consistency of the scale was excellent, with Cronbach’s α coefficients of 0.915 at Time 1 and 0.925 at Time 2. The four subscales also demonstrated satisfactory internal consistency, with Cronbach’s α coefficients ranging from 0.761 to 0.872.

#### 2.2.2. Measure of Psychological Resilience

Psychological resilience was assessed using the 10-item version of the Connor-Davidson Resilience Scale (CD-RISC-10), revised by Campbell-Sills and Stein (E. Wang et al., 2024). The CD-RISC-10 and RSQ have been used widely in recent digital-use research and shown adequate reliability across cultures and contexts; similarly, short-video specific scales (e.g., Short Video Addiction Scale) have displayed satisfactory internal consistency in university samples (Almulla et al., 2025). This instrument was adapted from the original 25-item CD-RISC and retains a unidimensional structure consisting of 10 items (e.g., “I am able to adapt to change,” “I can deal with whatever comes my way,” “I tend to bounce back after illness or hardship”). Each item was rated on a 6-point Likert scale ranging from 0 (“not true at all”) to 5 (“true nearly all the time”), with higher scores indicating greater levels of psychological resilience. Previous studies have demonstrated that the CD-RISC-10 possesses good reliability and validity across different cultural contexts and sample populations. In the present study, the scale demonstrated good internal consistency, with Cronbach’s α coefficients of 0.80 at Time 1 and 0.89 at Time 2.

#### 2.2.3. Measure of Rejection Sensitivity

Rejection sensitivity was assessed using the Rejection Sensitivity Questionnaire (RSQ) revised by Li Xia for use among Chinese populations (Yan & Ran, 2023). The questionnaire consists of 18 items (e.g., “I am sensitive to rejection,” “I am overly sensitive,” “I do not care much about whether others accept or reject me”), rated on a 5-point Likert scale ranging from 1 (“strongly disagree”) to 5 (“strongly agree”). Higher total scores indicate higher levels of rejection sensitivity. Previous studies have shown that the RSQ demonstrates good reliability and validity among Chinese adolescents and adults. In the present study, the scale demonstrated satisfactory internal consistency, with Cronbach’s α coefficients of 0.76 at Time 1 and 0.80 at Time 2.

### 2.3. Data Analysis

Data analyses primarily included preliminary data screening, common method bias assessment, descriptive statistics, Pearson correlation analyses, and cross-lagged panel model (CLPM) estimation. First, SPSS 21.0 was used to conduct preliminary data screening and descriptive statistical analyses, including assessments of normality and descriptive statistics for short-video addiction (SVA), psychological resilience (PR), and rejection sensitivity (RS). Second, Harman’s single-factor test was performed to assess the potential influence of common method bias arising from the use of self-report measures. Subsequently, Pearson correlation analyses were conducted to examine the bivariate associations among SVA, PR, and RS at both measurement waves. Finally, the cross-lagged panel model (CLPM) was estimated using R version 4.4.1 with the lavaan package(version 0.6-19) to examine the longitudinal associations among SVA, PR, and RS across the two measurement waves. In this model, SVA was specified as a latent construct with its four dimensions as indicators, whereas RS and PR were modeled as observed variables using their total scores, consistent with their conventional operationalization in previous research. This specification was based on theoretical considerations, as SVA represents a multidimensional behavioral construct, while RS and PR have been widely assessed using composite scores in previous research. Model fit was evaluated using the chi-square to degrees of freedom ratio (χ^2^/df), the Comparative Fit Index (CFI), the Tucker–Lewis Index (TLI), the Root Mean Square Error of Approximation (RMSEA), and the Standardized Root Mean Square Residual (SRMR). Statistical significance was set at *p* < 0.05.

## 3. Results

### 3.1. Method Bias Test

Because multiple self-report questionnaires were administered to the same group of participants, there was a potential risk of common method bias. To minimize such bias, participants were provided with standardized instructions and trained before data collection. Several procedural controls were implemented, including standardized guidance, strict time limits, alternating presentation of positively and negatively worded items, and inclusion of demographic variables as control factors. To statistically assess common method bias, all items from the SVA, SS, and PR scales were subjected to exploratory factor analysis using Harman’s single-factor test. Under unrotated conditions, ten factors with eigenvalues greater than 1 were extracted, with the first factor accounting for 20% of the total variance—well below the 40% threshold commonly used to indicate serious common method bias. Therefore, the results suggest that common method variance was not a major concern in this study.

### 3.2. Descriptive and Correlation Analysis

The results of the Pearson correlation analysis (see Table 1) showed that T1 SVA was significantly positively correlated with T2 SVA (r = 0.51, *p* < 0.01), and the correlation between T1 RS and T2 RS also reached a significant level (r = 0.51, *p* < 0.01). Similarly, T1 PR was significantly positively correlated with T2 PR (r = 0.33, *p* < 0.01). Furthermore, at the same measurement time point, T1 SVA was significantly correlated with both T1 RS and T1 PR (r = 0.42, *p* < 0.01 and r = −0.19, *p* < 0.01, respectively). The correlations between T2 SVA and T2 RS (r = 0.38, *p* < 0.01) and between T2 SVA and T2 PR (r = −0.21, *p* < 0.01) were also significant. These findings indicate that short-video addiction (SVA), psychological resilience (PR), and rejection sensitivity (RS) demonstrated stable longitudinal correlations and synchronous relationships across the two time points, meeting the statistical prerequisites for conducting cross-lagged analyses. Overall, the significant correlations among most core variables and their indicators provide empirical support for subsequent testing of mediation effects.

### 3.3. Cross-Lagged Analysis

**Figure 1:**
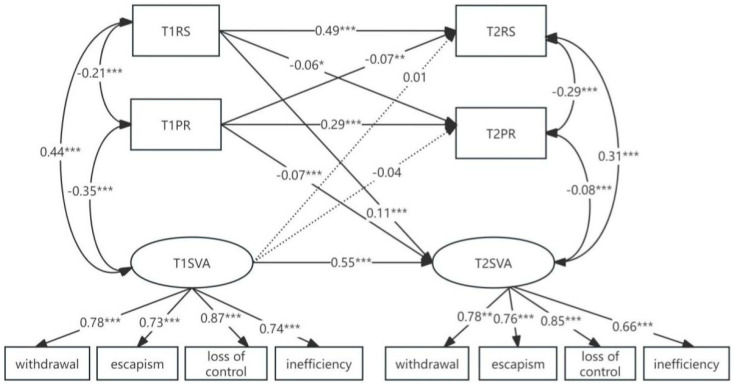
The results of the cross-lagged model analysis. Solid lines represent statistically significant paths, whereas dotted lines represent non-significant paths. Standardized coefficients are presented. * *p* < 0.05, ** *p* < 0.01, and *** *p* < 0.001.

To examine the longitudinal relationships among short-video addiction (SVA), psychological resilience (PR), and rejection sensitivity (RS) across different time points, a cross-lagged panel model (CLPM) was constructed (see Figure 1). The model demonstrated a good fit to the data: χ^2^/df = 4.52, CFI = 0.93, TLI = 0.91, RMSEA = 0.08, and SRMR = 0.07.

From T1 to T2, all autoregressive paths were significant: T1 RS → T2 RS (β = 0.49, *p* < 0.001), T1 PR → T2 PR (β = 0.29, *p* < 0.001), and T1 SVA → T2 SVA (β = 0.55, *p* < 0.001), indicating temporal stability of the three variables across the six-month period.

Regarding cross-lagged effects, the predictive paths from T1 SVA to T2 RS (β = 0.01, *p* > 0.05) and from T1 SVA to T2 PR (β = −0.04, *p* > 0.05) were nonsignificant. However, T1 RS negatively predicted T2 PR (β = −0.06, *p* < 0.05) and positively predicted T2 SVA (β = 0.11, *p* < 0.001). Likewise, T1 PR negatively predicted both T2 RS (β = −0.07, *p* < 0.05) and T2 SVA (β = −0.07, *p* < 0.05).

Concurrent correlations showed that at T1, RS was significantly negatively correlated with PR (β = −0.21, *p* < 0.001) and positively correlated with SVA (β = 0.44, *p* < 0.001), while PR was negatively correlated with SVA (β = −0.35, *p* < 0.001). At T2, RS and PR remained significantly negatively correlated (β = −0.29, *p* < 0.001), RS was positively correlated with SVA (β = 0.31, *p* < 0.001), and PR was negatively correlated with SVA (β = −0.08, *p* < 0.05).

Overall, SVA, RS, and PR exhibited temporal stability across the two waves. Reciprocal longitudinal associations were observed between rejection sensitivity and psychological resilience. In contrast, only rejection sensitivity and psychological resilience significantly predicted subsequent short-video addiction, whereas short-video addiction did not significantly predict subsequent rejection sensitivity or psychological resilience.

## 4. Discussion

### 4.1. Relationships Among Rejection Sensitivity, Psychological Resilience, and Short-Video Addiction

This study, employing a two-wave longitudinal design and cross-lagged modeling, systematically examined the longitudinal relationships among rejection sensitivity (RS), psychological resilience (PR), and short-video addiction (SVA). The findings indicated that bidirectional associations were evident between rejection sensitivity and psychological resilience, whereas the longitudinal associations involving short-video addiction were primarily unidirectional. The results showed that all three variables exhibited high stability over the six-month follow-up, indicating that short-video usage behavior and associated psychological traits tend to persist within a relatively short time interval. This finding aligns with prior research on the stability of addictive behaviors and individual psychological traits (Brand et al., 2019). Our longitudinal findings accord with updated interpretations of the I-PACE model, which posits that individual predispositions (e.g., rejection sensitivity) interact with affective and cognitive processes to produce maladaptive usage patterns, while psychological resources (e.g., resilience) may help attenuate these processes. Recent reviews re-emphasize this dynamic interplay and call for integrating neurobiological evidence with longitudinal behavioral data (Brand et al., 2025).

Longitudinal analyses revealed that RS may function as a vulnerability factor for SVA. Empirical research supports this mechanism: RS has been positively correlated with problematic Internet usage and negative psychosocial outcomes (e.g., depression, social anxiety, body image concerns) (Hawes et al., 2020; Y. Lin & Fan, 2023; Webb & Zimmer-Gembeck, 2016). High sensitivity to social threat can trigger negative emotions, which may increase the propensity to use short videos for immediate reward or emotional regulation and thereby elevate addiction risk (Brand et al., 2016, 2019). Short-video platforms, with algorithmic driving and instantaneous feedback, are particularly attractive to individuals with high RS. On one hand, mechanisms such as likes, comments, and shares can deliver rapid emotional reinforcement, causing attention bias for socially sensitive users and facilitating excessive use. On the other hand, algorithmic recommendation continuously feeds content highly aligned with users’ interests and emotional state, potentially amplifying immersive experiences and dependence for high RS individuals and intensifying compulsivity and addiction risk.

Recent studies further refine this pathway. For example, C. Xu et al. (2025) found among medical students that RS positively predicts loneliness and problematic internet use, with loneliness serving as a mediator and self-control moderating the latter stage of the indirect effect. This underscores the importance of loneliness and regulatory capacity as boundary conditions in the RS → problematic use pathway. Moreover, psychological resilience (PR) in this study demonstrated a protective role. This is consistent with prior findings that resilience can mitigate the risk of internet or mobile addiction by enhancing emotion regulation and adaptive coping (Chen et al., 2025; Cui & Chi, 2021; Liu et al., 2024). It also aligns with the theoretical positioning of resilience as a positive psychological resource (Fletcher & Sarkar, 2013). Individuals with higher resilience tend to employ more flexible and effective emotion regulation strategies, particularly favoring cognitive reappraisal over expressive suppression (Gross & John, 2003). Such proactive regulation can alleviate stress, anxiety, and boredom, thus reducing motivations to compensate via short-video engagement (Zhang et al., 2024). In doing so, resilience helps interrupt the “negative emotion → escapist use → addiction” feedback loop (Kardefelt-Winther, 2014).

Recent empirical work supports the mediating or buffering role of resilience in digital addiction contexts. For instance, Bağatarhan (2025) examined adolescents and found that resilience mediated the relationship between self-efficacy, happiness, social support, and internet addiction. This suggests resilience mitigates the influence of multiple psychosocial risk factors on problematic usage trajectories (Bağatarhan, 2025). Also, Almulla et al. (2025) in a cross-cultural sample observed that resilience is predictive of internet addictive behaviors, although the relationship can vary across cultural contexts, highlighting that resilience may operate differently depending on sociocultural norms.

From a cognitive and neurobehavioral perspective, resilient individuals often exhibit stronger executive functions (e.g., impulse inhibition, delayed gratification) (Diamond, 2013). Such individuals are better able to resist impulsive browsing of short videos and mitigate the allure of variable-ratio reinforcement designs (Clark & Zack, 2023). They also tend to display stronger self-monitoring and behavioral control (Duckworth & Gross, 2014), allowing them to set usage boundaries and adhere to them, thus reducing overuse risks. Resilience is also associated with problem-focused adaptive coping. Under stress and challenge, individuals higher in resilience are more likely to address problems proactively or seek social support rather than resorting to short-video escapism (Ye et al., 2020). This positive orientation helps avoid the vicious cycle of “stress → escapism → addiction → functional impairment → increased stress” (Brand et al., 2019).

Cross-sectional correlation analyses further indicated that rejection sensitivity (RS) and psychological resilience (PR) were significantly negatively correlated at both time points (T1 β = −0.21; T2 β = −0.29), RS was positively correlated with short-video addiction (SVA) (T1 β = 0.44; T2 β = 0.31), whereas PR was negatively correlated with SVA (T1 β = −0.35; T2 β = −0.08). This co-occurrence pattern suggests that individuals with high RS tend to exhibit lower PR and are more prone to short-video addiction, likely due to heightened sensitivity to social threats and a greater tendency toward negative emotional reactivity. Conversely, individuals with higher PR may mitigate addiction risk by employing more flexible and effective emotion regulation and adaptive coping strategies (Almulla et al., 2025; London et al., 2007; Önal & Özdemir, 2025; R. Wang et al., 2025). It should be noted that cross-sectional analyses reflect covariance rather than causal effects, and interpretation of these associations should be complemented with longitudinal findings. Longitudinal results indicated that RS positively predicted subsequent SVA. This association may be interpreted in light of theoretical accounts emphasizing emotion regulation and adaptive coping, although these mechanisms were not directly examined in the present study.PR was prospectively associated with lower subsequent SVA and may represent a protective psychological characteristic.

Theoretically, these findings are broadly consistent with a dual-pathway model: a risk pathway driven by RS, which increases short-video addiction through heightened negative affect and escapist motives; and a protective pathway driven by PR, and a protective pathway driven by PR, which may contribute to lower addiction tendencies, potentially through mechanisms such as emotion regulation, cognitive reappraisal, and problem-solving, as suggested by previous theoretical and empirical research. This “risk–protection” framework extends current behavioral addiction theories and provides empirical guidance for interventions, suggesting that strategies should simultaneously reduce individual vulnerability and enhance resilience to effectively prevent addiction.

Moreover, recent research indicates that PR is closely associated with executive functions and self-monitoring abilities (Diamond, 2013; Duckworth & Gross, 2014), which help individuals resist the variable-ratio reinforcement mechanisms embedded in short-video platforms (Montag et al., 2018). Thus, high-resilience individuals are not only psychologically protected but also behaviorally better equipped to regulate their media use. Based on previous theoretical and empirical literature, interventions targeting cognitive control, emotion regulation, and behavioral self-monitoring may represent potential directions for future prevention.

### 4.2. Rejection Sensitivity Influences Short-Video Addiction via Psychological Resilience: An I-PACE Model Perspective

Consistent with the I-PACE model (Brand et al., 2016, 2019), The present findings suggest that rejection sensitivity (RS) may represent a vulnerability factor associated with subsequent short-video addiction, partly through its negative association with psychological resilience (PR). Individuals high in RS tend to perceive social interactions as threatening, depleting emotional resources and weakening resilience—the capacity to regulate negative emotions and cope with stress and setbacks (Hou et al., 2019; Ma et al., 2022). Reduced resilience, in turn, impairs adaptive coping and promotes compensatory short-video use for immediate emotional relief. These results empirically support the I-PACE framework, providing evidence that individual predispositions may influence addictive behaviors through affective and cognitive regulation processes, while resilience serves as a protective buffer within this pathway.

Further, our study demonstrates a bidirectional predictive relationship between RS and PR. On one hand, individuals with high RS are more likely to interpret ambiguous or neutral social cues as rejection or negative feedback, accumulating negative emotional experiences, depleting psychological resources, and thereby reducing PR (Kökönyei et al., 2024; Shan et al., 2021). On the other hand, PR, as a positive protective factor, can alleviate negative emotional responses caused by social sensitivity by enhancing emotion regulation, promoting cognitive reappraisal, and facilitating self-monitoring, thereby reducing overreactions and impulsive behaviors in social contexts (Liang et al., 2021; Önal & Özdemir, 2025). Therefore, RS and PR may interact through a vicious cycle of “heightened sensitivity → resource depletion → decreased resilience” and a virtuous cycle of “enhanced resilience → positive regulation → reduced sensitivity,” jointly influencing the onset and development of short-video addiction (Brand et al., 2019).

From behavioral and cognitive perspectives, individuals with higher PR generally exhibit stronger executive functions, self-monitoring, and impulse control (Diamond, 2013; Duckworth & Gross, 2014). These abilities may help individuals to resist addictive mechanisms in short-video platforms based on variable-ratio reinforcement (Montag et al., 2018), establish reasonable usage boundaries, and adhere to them, reducing the risk of overuse. Moreover, highly resilient individuals tend to adopt problem-focused coping strategies, proactively seeking resources and social support rather than resorting to short-video escapism (Sun et al., 2024; Y. Xu et al., 2023; Zhao & Kou, 2024). Taken together with previous research, this indicates that PR provides protection not only at the emotional level but also at the behavioral level, mitigating addiction tendencies.

In summary, this study suggests a differentiated pattern of longitudinal associations among RS, PR, and SVA: RS increases the likelihood of short-video dependence by enhancing sensitivity to negative emotions and interpersonal threats; PR may be associated with lower SVA, potentially through mechanisms proposed in previous theoretical and empirical research, such as emotion regulation, executive functioning, and behavioral self-control. These findings support the dual role of psychological vulnerability and psychological resources in the formation of addictive behaviors and provide theoretical guidance for the prevention and intervention of short-video addiction (Belfiore et al., 2024; C. Lin et al., 2024). Emerging neuroimaging studies indicate that excessive short-video use is associated with functional and structural changes in reward-related and executive control brain regions, reduced loss aversion, and impaired evidence accumulation during decision tasks, which may help explain increased impulsivity and risk-taking observed behaviorally. These neurobehavioral findings strengthen the view that short-video overuse can engage mechanisms similar to other behavioural addictions (Almulla et al., 2025). Interventions may benefit from adopting a holistic I-PACE perspective, targeting both the reduction in RS and social vulnerability and the enhancement of PR through emotion regulation training, cognitive reappraisal skill development, and improvement of real-life social skills, thereby interrupting the vicious cycle of “sensitivity → resource depletion → addiction” and reducing short-video addiction risk among adolescents and young adults (Brand et al., 2019; Taş, 2019).

### 4.3. Limitations and Future Research Implications

This study employed a cross-lagged panel model to examine the dynamic relationships among psychological resilience (PR), rejection sensitivity (RS), and short-video addiction (SVA), revealing the interaction patterns of these variables over time. However, several limitations should be addressed in future research.

First, the study’s time span was six months, which allowed for some reflection of the longitudinal influence among variables but was insufficient to capture the dynamic evolution of these constructs over longer periods. In particular, stable psychological traits such as PR and RS may exhibit developmental trajectories over multiple years. Therefore, future research should extend the follow-up period, collecting longitudinal data spanning at least three years, enhancing the robustness of longitudinal interpretations and further verifying the stability of directional effects and effect sizes between variables.

Second, the sample was relatively concentrated, potentially limiting the generalizability of the findings. Future studies could conduct cross-cultural and cross-population validations across different regions, cultural backgrounds, and age groups to examine the universality of the model and potential cultural variations. Additionally, this study primarily relied on self-report questionnaires. Although measurement error was controlled, self-report data are inevitably subject to subjective bias. Future research should combine longitudinal self-report with objective behavioral logs, ecological momentary assessment (EMA), and neuroimaging or psychophysiological measures to better understand potential causal pathways; advanced longitudinal methods such as cross-lagged panel network analysis or random-intercept CLPM are recommended to distinguish within-person effects from between-person stability.

Finally, while the current model included three core psychological and behavioral variables, other potential mediators or moderators may have been omitted for example, social support, academic stress, and emotion regulation strategies which might further explain the complex mechanisms linking SVA, PR, and RS, though these possibilities remain to be empirically validated. Future studies could employ multilevel models or structural equation models to comprehensively examine the pathways at individual, contextual, and cultural levels.

Although several cross-lagged paths reached statistical significance, the standardized regression coefficients were relatively modest. Given the relatively large sample size, statistically significant effects should not be interpreted as indicating strong longitudinal relationships or substantial practical significance. Instead, the present findings suggest modest prospective associations among psychological resilience, rejection sensitivity, and short-video addiction that are broadly consistent with the theoretical framework while warranting cautious interpretation. In addition, the autoregressive coefficients indicated that psychological resilience and rejection sensitivity exhibited only modest temporal stability across the six-month interval, particularly for rejection sensitivity. Although rejection sensitivity has often been conceptualized as a relatively stable psychological characteristic, the present findings suggest that both constructs retained meaningful variability over time. Therefore, the observed cross-lagged associations should be interpreted in light of both the magnitude of the effects and the temporal stability of the constructs. Future research should examine whether these associations replicate across longer follow-up periods and more diverse populations.

Overall, this study contributes to understanding the longitudinal interplay between psychological resources and personality traits in digital media use. It suggests theoretical and practical implications for developing interventions aimed at reducing short-video addiction, enhancing psychological resilience, and decreasing rejection sensitivity. Future research should continue to refine study samples, measurement methods, and model construction to develop more targeted and generalizable strategies for promoting mental health and guiding healthy media use.

## 5. Conclusions

Based on the I-PACE model, the present study suggested that rejection sensitivity, psychological resilience, and short-video addiction were longitudinally associated over time, with bidirectional associations observed between rejection sensitivity and psychological resilience, whereas short-video addiction was primarily predicted by these psychological factors rather than prospectively predicting them. Specifically, rejection sensitivity was prospectively associated with higher levels of short-video addiction, whereas psychological resilience was prospectively associated with lower levels of short-video addiction. Although the observed longitudinal effects were modest, they are consistent with the proposed theoretical framework and highlight potential longitudinal relationships that warrant further investigation. These findings underscore the importance of considering both risk and protective factors within an integrative framework when examining the psychological mechanisms underlying short-video addiction. Furthermore, the results suggest potential implications for intervention, indicating that approaches aimed at reducing rejection sensitivity and enhancing psychological resilience—such as emotion regulation training, cognitive reappraisal, and social skills enhancement—may be considered potential targets for future preventive interventions aimed at reducing short-video addiction among young adults.

## Figures and Tables

**Table 1:** Descriptive statistics and correlation analysis results (*n* = 1394).

Variable	M	SD	1	2	3	4	5	6	7	8	9
age	19.49	0.92	1								
sex	1.70	0.46	−0.03	1							
Only child	1.85	0.36	0.04	0.14 **	1						
T1SVA	36.53	10.25	−0.01	0.17 **	0.04	1					
T1RS	53.99	8.86	−0.01	0.20 **	−0.01	0.42 **	1				
T1PR	35.56	6.51	−0.01	−0.05	0.02	−0.19 **	−0.35 **	1			
T2SVA	36.27	10.30	0.02	0.10 **	0.08 **	0.51 **	0.26 **	−0.09 **	1		
T2RS	54.63	8.54	−0.04	0.14 **	0.04	0.23 **	0.51 **	−0.24 **	0.38 **	1	
T2PR	34.77	6.57	−0.01	−0.05 *	−0.06 *	−0.13 **	−0.20 **	0.33 **	−0.21 **	−0.34 **	1

Note. * *p* < 0.05, ** *p* < 0.01.

## Data Availability

The data that support the findings of this study are available on reasonable request from the corresponding author. The data are not publicly available due to privacy or ethical restrictions.

## Abbreviations

PS	Psychological resilience
RS	Rejection sensitivity
SVD	Short-video addiction
CD-RISC-10	Connor-Davidson Resilience Scale
RSQ	Rejection Sensitivity Questionnaire
CFI	Comparative fit index
TLI	Tucker–Lewis index
RMSEA	Root mean square error of approximation Standard
SRMR	Stand root mean square
SEM	Structural equation modeling
